# Automated acquisition of top-view dairy cow depth image data using an RGB-D sensor camera

**DOI:** 10.1093/tas/txac163

**Published:** 2022-12-13

**Authors:** Robert Kadlec, Sam Indest, Kayla Castro, Shayan Waqar, Leticia M Campos, Sabrina T Amorim, Ye Bi, Mark D Hanigan, Gota Morota

**Affiliations:** Department of Industrial and Systems Engineering, Virginia Polytechnic Institute and State University, Blacksburg, VA, USA; Department of Industrial and Systems Engineering, Virginia Polytechnic Institute and State University, Blacksburg, VA, USA; Department of Industrial and Systems Engineering, Virginia Polytechnic Institute and State University, Blacksburg, VA, USA; Department of Industrial and Systems Engineering, Virginia Polytechnic Institute and State University, Blacksburg, VA, USA; School of Animal Sciences, Virginia Polytechnic Institute and State University, Blacksburg, VA, USA; School of Animal Sciences, Virginia Polytechnic Institute and State University, Blacksburg, VA, USA; School of Animal Sciences, Virginia Polytechnic Institute and State University, Blacksburg, VA, USA; School of Animal Sciences, Virginia Polytechnic Institute and State University, Blacksburg, VA, USA; School of Animal Sciences, Virginia Polytechnic Institute and State University, Blacksburg, VA, USA; Center for Advanced Innovation in Agriculture, Virginia Polytechnic Institute and State University, Blacksburg, VA, USA

**Keywords:** depth data, image processing, precision livestock farming, sensor camera

## Abstract

Animal dimensions are essential indicators for monitoring their growth rate, diet efficiency, and health status. A computer vision system is a recently emerging precision livestock farming technology that overcomes the previously unresolved challenges pertaining to labor and cost. Depth sensor cameras can be used to estimate the depth or height of an animal, in addition to two-dimensional information. Collecting top-view depth images is common in evaluating body mass or conformational traits in livestock species. However, in the depth image data acquisition process, manual interventions are involved in controlling a camera from a laptop or where detailed steps for automated data collection are not documented. Furthermore, open-source image data acquisition implementations are rarely available. The objective of this study was to 1) investigate the utility of automated top-view dairy cow depth data collection methods using picture- and video-based methods, 2) evaluate the performance of an infrared cut lens, 3) and make the source code available. Both methods can automatically perform animal detection, trigger recording, capture depth data, and terminate recording for individual animals. The picture-based method takes only a predetermined number of images whereas the video-based method uses a sequence of frames as a video. For the picture-based method, we evaluated 3- and 10-picture approaches. The depth sensor camera was mounted 2.75 m above-the-ground over a walk-through scale between the milking parlor and the free-stall barn. A total of 150 Holstein and 100 Jersey cows were evaluated. A pixel location where the depth was monitored was set up as a point of interest. More than 89% of cows were successfully captured using both picture- and video-based methods. The success rates of the picture- and video-based methods further improved to 92% and 98%, respectively, when combined with an infrared cut lens. Although both the picture-based method with 10 pictures and the video-based method yielded accurate results for collecting depth data on cows, the former was more efficient in terms of data storage. The current study demonstrates automated depth data collection frameworks and a Python implementation available to the community, which can help facilitate the deployment of computer vision systems for dairy cows.

## INTRODUCTION

Tracking changes in animal dimensions, such as body mass, body conformation, and body condition score, can provide insights into factors affecting growth rates, market weight, diet efficiency, energy balance, and health status (Tscharke and Banhazi, 2013). Managing gain and loss of body mass are critical for circumventing production loss, inefficient feeding, poor reproductive performance, and adverse health events. Therefore, regularly monitoring body weight or body condition scores of animals throughout their lifespan is vital. However, current collection methods involve manual manipulation of the animals which prevents mass application of the technique. For example, the challenges in obtaining regular weights of animals have driven most operations to weigh animals only a few times during their productive lifespan (e.g., birth, weaning, and finishing) or to rely on more subjective measures of body fatness such as condition scores. This practice creates a paucity of data and prevents a robust understanding of the true growth curves and body fat reserves of animals. This bottleneck limits the scale and throughput of phenotyping activities and prevents comprehensive characterization of animals at an individual level.

Precision livestock farming or smart farming uses sensing technology to capture morphometric changes in animal growth and body composition dynamics. This technology optimizes production while ensuring sustainability and welfare (Morota et al., 2018). Computer vision systems provide a noninvasive method to efficiently measure the physical status of animals, such as body weight and body condition score. Recently, low-cost 3D depth sensor cameras, also known as RGB-D cameras, have become available to provide additional depth information (i.e., height at various points of interest) using infrared sensors that facilitate close monitoring of animals. Top-view depth images are collected to evaluate the body weight or morphometric traits of beef cattle (Gomes et al., 2016; Cominotte et al., 2020; Kamchen et al., 2021), dairy cattle (Spoliansky et al., 2016; Nir et al., 2018; Song et al., 2018), sheep (Samperio et al., 2021), and swine (Fernandes et al., 2019; Condotta et al., 2020; Yu et al., 2021). The overhead camera position is suitable because it is noninvasive and does not interfere with the daily farm operations. However, the cameras are generally manually controlled through a laptop, and automated image data acquisition procedures are not well documented in the literature. Furthermore, open-source computer code is rarely available even for noncommercial image acquisition systems. Continuous recording of depth images generates a massive amount of data leading to storage and transfer problems. To fully automate the depth data acquisition process for each animal, a computer vision system should automatically detect the animal after it comes into the camera frame, trigger recording, capture depth data, and stop recording once the animal is out of the frame. The objective of this study was to 1) investigate the utility of two automated top-view depth data acquisition methods (picture and video) using dairy cows, 2) evaluate the performance of an infrared cut lens, and 3) to make open-source implementations available to the animal science community.

## MATERIALS AND METHODS

### Animals

Animal handling and media recording were approved and carried out in accordance with the Virginia Tech Institutional Animal Care and Use Committee (IACUC). In total, 150 Holstein and 100 Jersey animals at the Kentland Farm Dairy Complex (Virginia Tech, Blacksburg, VA) were available during the study. The Holsteins were approximately 2 years old, and the Jerseys ranged from 2 to 6 years. These cows averaged 190 ± 111 days in milk and 665 ± 124 kg body weight. Cows were housed in a free-stall barn, milked twice per day (lactating cows), fed ad libitum once per day, and had free access to water. Data collection occurred in October and November 2021 after cows exited the milking parlor from the 12 PM milking session.

### Depth Sensing System

**Figure 1.**
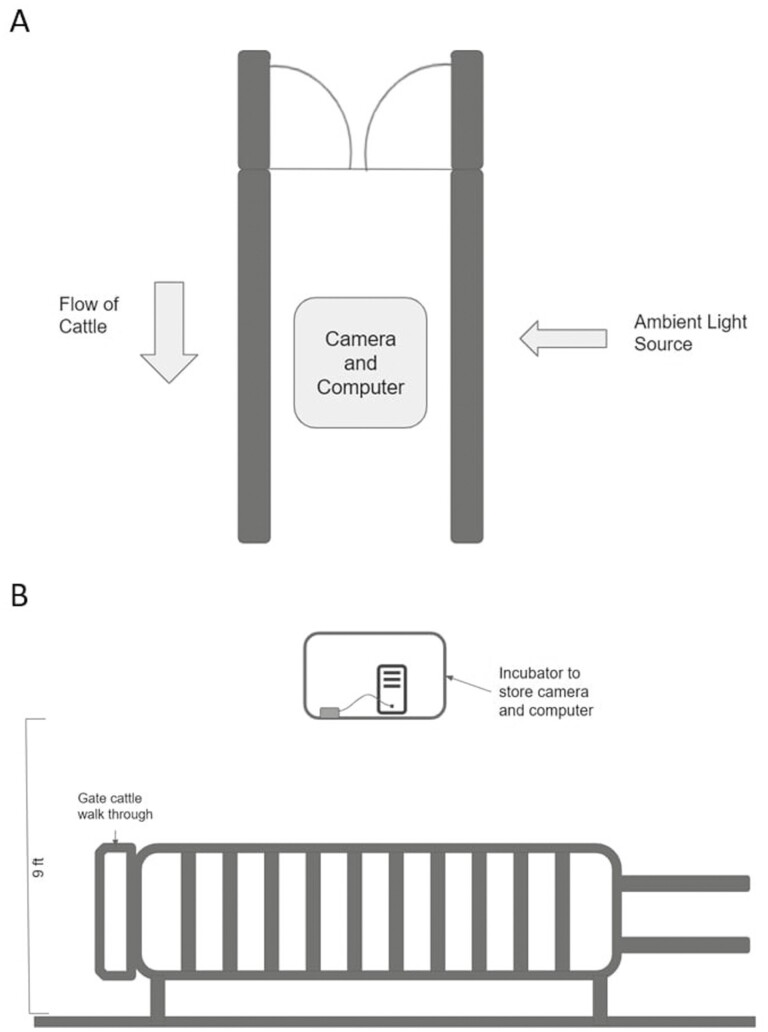
Schematic representation of the depth data acquisition system from A) top and B) side views. The depth camera was positioned above the walk-through path.

An Intel RealSense D435 depth sensor camera (Intel, Santa Clara, CA, USA) was used to collect depth data. It provides 87° horizontal and 58° vertical field of view. Under ideal lighting conditions, the camera uses two stereos to determine the depth (Intel RealSense, 2022). As the distance between the two stereo image sensors is known, the camera can compare the two images taken to determine the depth. This is the same principal as humans using two eyes to gauge depth. Under poor lighting conditions, the camera uses an infrared dot pattern. Because the projected pattern is known, the camera can measure the distances between dots to determine the depth. In our experiment, lighting was provided both naturally and artificially (electric light). Stereo image sensors were used to measure the depth because the testing environment had good light conditions. The sensor camera was mounted in a heated container to maintain camera temperatures within the normal operating range (0 °C to 35 °C). The container was mounted 2.75 m above a one-way exit lane between the milking parlor and pen housing, and positioned straight down to collect top-views of cows walking under it. This design allowed a cow to voluntarily walk below the camera in an unconstrained manner after exiting the milking parlor. The top- and side-view diagrams of the imaging system are shown in Figure 1. The path was sufficiently narrow to limit traffic to a single cow at a time. The lane was fitted with a weight-activated door that prevented multiple cows from entering simultaneously. A laptop was connected to the depth-sensing camera using USB 3.1 cable. The camera used auto-exposure and auto focus.

### Depth Image Data Acquisition

Picture- and video-based methods that automatically detect and capture depth data were investigated. Both methods were implemented in Python using the pyrealsense2 library provided by Intel RealSense. The scripts were run on the computer by setting the resolution to 1,280 × 720 pixels, such that the head-to-tail of the cow was captured by the 1,280 pixels and the width of the cow by 720 pixels. The 1280-pixel width enclosed the entire length of the walk-through area. Pictures and videos were saved in a.bag format. The same camera setup was used for both the picture and video collections. The laptop used was a Microsoft Surface Book 2. The computer had 16GB of RAM, 512GB SSD, Intel Core i7, and an NVIDIA GeForce GTX 1050 graphics card. The testing of both scripts occurred between 12 pm and 3 pm. The cows traveled from the milking parlor through the camera setup in a single line. The speeds at which the cows entered the walk-through path varied between a slow walk and a mild trot.

#### Picture-based method.

**Figure 2.**
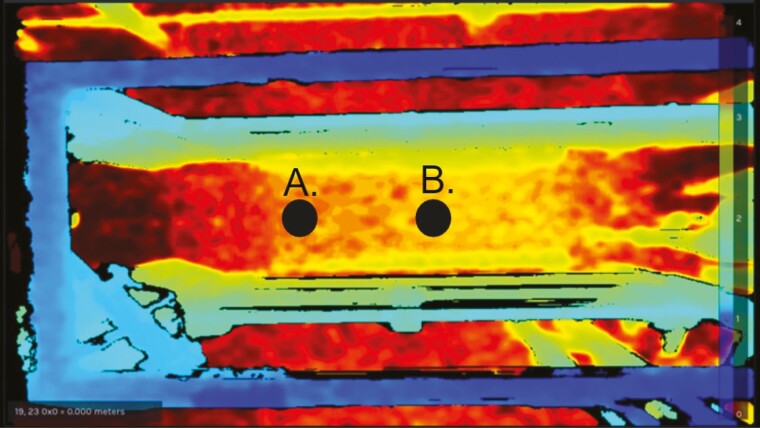
Locations of point-of-interests (black points) in depth data for A) picture- and B) video-based methods. The point-of-interests were enlarged for a visualization purpose. Depth information is indicated with the level of brightness.

This method collected a predetermined number of images. Images were taken using the pyrealsnese2 library (pyrealsense2.save_single_frameset function). The advantage of this method is that it reduces the total file size and saves disk space. A point-of-interest (POI) was used for both methods (Figure 2). A POI is a pixel location at which the depth is monitored. The picture-based POI was set 1.52 m from the entrance of the imaging system. When an object passed through the POI, a set of 3 or 10 pictures was taken at intervals of 0.75 or.15 seconds, respectively. Once the set of pictures was collected, no more pictures were taken until the current cow left the POI and a new one entered. Once the pictures were taken, they were saved locally using a timestamp as the file name.

#### Video-based method.

The video-based method collected a sequence of images as a video. The number of frames per second was set to 30. The video-based POI was set roughly 0.61 m from the entrance, which allowed sufficient time for the camera to begin recording and limited the number of false recordings caused by cows lingering in the walk-through area. The video-based method started recording when the distance to the POI changed by 0.6 m (signaling that an object had entered the frame) and stopped recording when the distance to the POI returned to the distance to the floor. The recordings were saved locally using a timestamp as the file name.

### Infrared Cut Lens

To reduce errors in both the picture- and video-based methods, we also explored the use of an infrared cut lens (Quanmin Ltd., China) attached to the camera. Image capture methods remained unchanged; the only variable added was the lens. The goal of this procedure was to reduce the ambient light reaching the camera allowing the camera to obtain better readings using the infrared dots being projected.

### Method Evaluation

Before applying image segmentation, all videos and pictures were passed through rs-convert (Intel RealSense, 2021), an open-source program that converts.bag files to.PNG. OpenCV was used in Python to extract the cows from the depth images. The boundaries in the vertical direction were defined as the fence rails. First, the image was cropped to remove the surrounding area, while leaving the walk-through area intact. The cropped image was then converted into a hue, saturation, and value image. The hue value was converted into a threshold image. Image contours were detected by extracting the x and y coordinates of the contour of the threshold image. The largest contour is maintained for the final image result. Morphological closing was used to fill the small, empty sections within the retained contour using structural elements of square 10 × 10.

Picture- and video-based methods were evaluated for the quality of depth data on an individual cow and a per-image basis. At the cow level, capturing at least two images was considered a success per visit on a given day. Each method was considered successful if the postsegmentation image was sufficient to determine the height, width, and length of the cow. Images were considered unsuccessful if the height, width, or length of the cow could not be accurately determined. The Python code used to capture automated depth data is available on GitHub (https://github.com/codeandstuf/CattleDepthCollection) as a freely downloadable package and the source code is provided as open source.

## RESULTS

**Figure 3.**
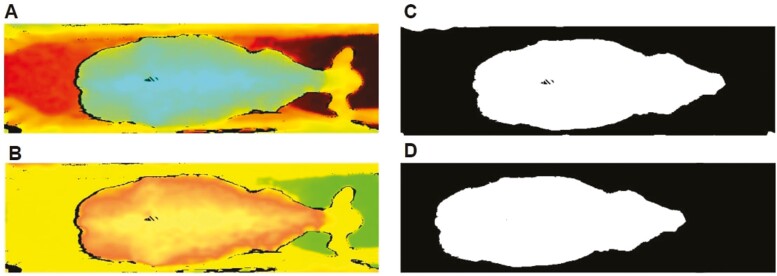
Example of image segmentation steps. A) Cropped depth picture taken using the D435 camera. B) Hue, saturation, value image. C) Threshold image. D) Final result. The largest contour was kept, and morphological closing was used to fill small empty areas within contour. Depth information is indicated with the level of brightness.

**Figure 4.**
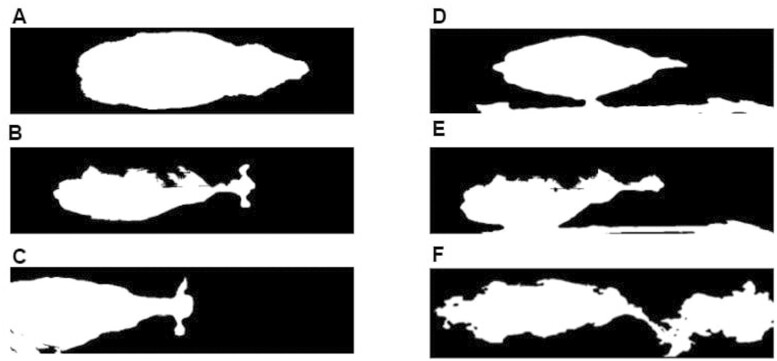
Examples of postsegmented depth image data. A) Successful image. B) Unsuccessful image due to quality. C) Unsuccessful image due to the cow not being in the field of vision. D) Unsuccessful image due rail separation. E) Unsuccessful image due to quality and rail separation. F) Unsuccessful image due obstructed view from multiple cows in the same image.

Each image collected from the picture- and video-based methods underwent a series of image-processing procedures (Figure 3). Examples of successful and unsuccessful images after image segmentation and their causes are shown in Figure 4. Each image was classified into six categories: successful image, unsuccessful image due to quality, unsuccessful image due to the cow not being in the field of vision, unsuccessful image due to rail separation, unsuccessful image due to quality and rail separation, and unsuccessful image due to an obstructed view from multiple cows in the same image.

### Picture-based Method

A picture-based method with three pictures was evaluated using 237 cows. Of the 237 tested, 165 were evaluated as successful (Figure 4A), resulting in an overall success rate of 69.62% (Table 1). For the 237 cows captured, 711 images were obtained. Analyzing the picture-based results on a per-image level, 50.63% of the images were successful. The remaining 49.37% of the images were considered unsuccessful with two main causes being poor quality (Figure 4B) and the cow being partially out of image (Figure 4C). This was followed by the fence rail not being completely separated from the cow (Figures 4D and E). The majority of cows were captured in the second picture of the three-picture series. Cows that moved slower than average were usually captured in the third image and cows moving faster than average were captured in the first image. The missing data chunks were mainly located on the left side of the cow, which was lit by ambient light (Figure 4B). Overexposed portions of cows resulted in poor depth readings. Cows with light-colored portions, including tan on Jerseys and white on Holsteins, were affected more by missing data chunks than the cows with dark-colored spots.

**Table 1.** Success rate of picture- and video-based methods per cow

Lenses^*a*^	Method	Cows tested	Number successful	Number unsuccessful	% Successful
Without	Picture (3)^*b*^	237	165	72	69.62%
	Picture (10)^*c*^	202	183	19	90.59%
	Video	203	181	22	89.16%
With	Picture (3)^*b*^	72	47	25	65.28%
	Picture (10)^*c*^	88	81	7	92%
	Video	62	61	1	98.39%

^*a*^Infrared cut lenses.

^*b*^Picture-based method with 3 pictures.

^*c*^Picture-based method with 10 pictures.

A picture-based method using 10 pictures was evaluated with 202 cows. Of the 202 tested, 183 were evaluated as successful (Figure 4A), resulting in an overall success rate of 90.59% (Table 1). For the 202 captured cows, 2,020 images were captured. At the per-image level, 47.03% of the images were successful. The remaining 52.97% of images were considered unsuccessful with the two main causes being poor quality (Figure 4B) and the cow being partially out of image (Figure 4C). Overall, the same trend was observed for the picture-based method with three pictures; however, the percentage of out-of-frame images increased in the picture-based method with 10 pictures. Additionally, the percentage of images with rails that were not completely separated from the cow decreased. A few images included images of multiple cows.

### Video-based Method

A video-based method was evaluated using 203 cow passes. Of the 203 tested, 181 were evaluated as successful, resulting in an overall success rate of 89.16% (Table 1). Capturing of 203 cows resulted in 19,184 frames. In total, 24.3% of the frames were graded as successful (Figure 4A), and 75.7% were graded as unsuccessful. The most common causes for unsuccessful frames were the cow being out of frame (Figure 4C) and poor image quality (Figure 4B). The majority of the frames containing a partial view of the cow was the result of camera starting a recording before the cow was fully in view. The cause of poor image quality was the same as previously mentioned in the picture-based method; excessive ambient lighting affected lighter-colored cows, specifically Jersey cows, and the white areas on Holstein cows. Multiple cows in the walk-through area were recorded only when the workers were near the imaging system. The best results were obtained when no humans were present in view of the imaging system.

### Infrared Cut Lens

Using the infrared cut lens, 72 cows were tested using the picture-based method with three pictures, 88 cows were tested using the picture-based method with 10 pictures, and 62 were tested using the video-based method. The application of the infrared cut lens resulted in success rates of 65.28%, 92.00%, and 98.39% for the picture-based method with three pictures, 10 pictures, and video-based methods, respectively (Table 1). The picture-based method with three and ten pictures had image success rates of 42.13% and 43.75%, respectively. The main cause of failure was the cows being out of image. The video-based method had a frame success rate of 44.95%. Similar to the picture-based method, the largest cause of failure was the cows being out of frame.

## DISCUSSION

With the development of high-throughput technologies, animal science has become a large-scale, data-rich field (Morota et al., 2021). Reducing labor costs and enhancing welfare are the primary goals that promote livestock productivity and operational sustainability. Computer vision or image-based phenotyping can be used to obtain morphometric measurements of animals using a top-view depth sensor camera. The process involves automation of depth-image acquisition and automation of image processing. Although the former tends to receive less attention than the latter in the literature, both types of automation are required to fully deploy computer vision systems on a farm. In this study, we evaluated the utility of two methods for automatically collecting dairy cow top-view depth data. On an individual cow basis, images were successfully captured for more than 89% of the cows using both picture- and video-based methods. The success rate further improved to 98% when the video-based method was combined with the infrared cut lens. Because at least one image is required from each cow to perform image processing analyses, this can be considered satisfactory. The video-based method had a higher success rate in capturing more useful depth frames than the picture-based method using three pictures. However, by increasing the number of pictures taken, the picture-based method with ten pictures achieved a comparable or slightly better success rate than the video-based method. The largest drawback of the video-based method is its large storage space requirement.

The 237 cows recorded using the picture-based method with three pictures consumed 476.47 MB of storage space with average storage space per cow being 2.01 MB. The picture-based method with ten pictures consumed 1.35 GB, with average storage space per cow being 6.7 MB. The 203 cattle recorded using the video-based method required 11,530.4 MB of storage space with average storage space per cow being 56.83 MB. The large storage space requirement combined with 47.1% of the frames containing out-of-view cows suggests that this method can be improved further. Two improvements proposed to reduce the number of unsuccessful frames are starting the recordings when the cow is further on the POI (e.g., between points A and B in Figure 2) and limiting the recordings to five seconds. Implementing these two improvements would reduce the number of frames captured, resulting in reduced total frames being stored, and thus fewer storage requirements.

The main cause of failure to record a successful image of a cow was poor quality. Although the largest portion of failed images were cows out of frame, this rarely did not prevent a usable image from being collected, as multiple images were taken in both collection methods. There were cases in which the camera was unable to collect any usable images because of poor quality. The main cause of image quality issues was the ambient lighting. An infrared cut lens was added to reduce failures owing to poor quality. At the cow level, the use of an infrared cut lens increased the success rate of the picture-based method with 10 pictures and the video-based method. On a per-image basis, failure due to poor quality was also reduced. Without further testing, no solid conclusion could be drawn regarding the effect of the lens. However, our results suggest that the lens improved the image quality for the picture-based method with 10 pictures and the video-based method.

The video-based method can potentially be improved by moving the POI further into the walk-through area and setting a maximum video length of five seconds. Moving the POI decreases the number of frames in which the cow is not fully in view of the camera. Reducing the frames where the cow was out of view would decrease storage requirements. Setting the maximum video length would also reduce storage requirements by preventing longer videos caused by cattle remaining stationary in the area.

In summary, we evaluated picture- and video-based methods that can automatically capture dairy cow depth data only when each cow was present within the data acquisition system. These methods do not require manual intervention or continuous recording of a video when a cow is not under the data acquisition system. Although the performances of the picture-based method with 10 pictures and the video-based method are comparable, the picture-based method is more efficient in terms of data storage. Cow identification can be assigned to each image through integration of information from a radio-frequency identification reader. Python implementations of the picture- and video-based methods were provided as free and open sources. We contend that detailed descriptions of automated depth data acquisition methods and the availability of Python implementations can facilitate the deployment of computer vision systems in livestock species.

## References

[CIT0001] Cominotte, A., A. Fernandes, J. Dorea, G. Rosa, M. Ladeira, E. van Cleef, G. Pereira, W. Baldassini, and O. M. Neto. 2020. Automated computer vision system to predict body weight and average daily gain in beef cattle during growing and finishing phases. Livest. Sci. 232:103904. doi:10.1016/j.livsci.2019.103904

[CIT0002] Condotta, I. C., T. M. Brown-Brandl, S. K. Pitla, J. P. Stinn, and K. O. Silva-Miranda. 2020. Evaluation of low-cost depth cameras for agricultural applications. Comput. Electron. Agric. 173:105394. doi:10.1016/j.compag.2020.105394

[CIT0003] Fernandes, A. F., J. R. Drea, R. Fitzgerald, W. Herring, and G. J. Rosa. 2019. A novel automated system to acquire biometric and morphological measurements and predict body weight of pigs via 3D computer vision. J. Anim. Sci. 97:496–508. doi:10.1093/jas/sky418 30371785 PMC6313152

[CIT0004] Gomes, R., G. Monteiro, G. Assis, K. Busato, M. Ladeira, and M. Chizzotti. 2016. Estimating body weight and body composition of beef cattle trough digital image analysis. J. Anim. Sci. 94:5414–5422. doi:10.2527/jas.2016-0797 28046161

[CIT0005] Intel RealSense. 2022. Beginner’s guide to depth (updated). [accessed March 6, 2022]. https://www.intelrealsense.com/beginners-guide-to-depth.

[CIT0006] Intel RealSense. 2021. Intel RealSense GitHub. [accessed December 1, 2021]. https://github.com/IntelRealSense.

[CIT0007] Kamchen, S. G., E. F. dos Santos, L. B. Lopes, L. G. Vendrusculo, and I. C. Condotta. 2021. Application of depth sensor to estimate body mass and morphometric assessment in Nellore heifers. Livest. Sci. 245:104442. doi:10.1016/j.livsci.2021.104442

[CIT0008] Morota, G., H. Cheng, D. Cook, and E. Tanaka. 2021. ASAS-NANP Symposium: prospects for interactive and dynamic graphics in the era of data-rich animal science. J. Anim. Sci. 99:skaa402. doi:10.1093/jas/skaa402 33626150 PMC7904041

[CIT0009] Morota, G., R. V. Ventura, F. F. Silva, M. Koyama, and S. C. Fernando. 2018. Big data analytics and precision animal agriculture symposium: machine learning and data mining advance predictive big data analysis in precision animal agriculture. J. Anim. Sci. 96:1540–1550. doi:10.1093/jas/sky014 29385611 PMC6140937

[CIT0010] Nir, O., Y. Parmet, D. Werner, G. Adin, and I. Halachmi. 2018. 3D computer-vision system for automatically estimating heifer height and body mass. Biosyst. Eng. 173:4–10. doi:10.1016/j.biosystemseng.2017.11.014

[CIT0011] Samperio, E., I. Lid´ on, R. Rebollar, M. Castej´ on-Limas, and C. Alvarez-Aparicio. 2021. Lambs’ live weight estimation using 3d images. Animal. 15:100212. doi:10.1016/j.animal.2021.100212 34029788

[CIT0012] Song, X., E. Bokkers, P. van der Tol, P. G. Koerkamp, and S. Van Mourik. 2018. Automated body weight prediction of dairy cows using 3-dimensional vision. J. Dairy Sci. 101:4448–4459. doi:10.3168/jds.2017-13094 29477535

[CIT0013] Spoliansky, R., Y. Edan, Y. Parmet, and I. Halachmi. 2016. Development of automatic body condition scoring using a low-cost 3-dimensional Kinect camera. J. Dairy Sci. 99:7714–7725. doi:10.3168/jds.2015-10607 27320661

[CIT0014] Tscharke, M., and T. Banhazi. 2013. Review of methods to determine weight and size of livestock from images. Aust. J. Multi-Discipl. Eng. 10:1–17. doi:10.7158/14488388.2013.11464860

[CIT0015] Yu, H., K. Lee, and G. Morota. 2021. Forecasting dynamic body weight of nonrestrained pigs from images using an RGB-D sensor camera. Transl. Anim. Sci. 5:txab006. doi:10.1093/tas/txab006 33659861 PMC7906448

